# Pharmacogenomic testing for antidepressant treatment selection: lessons learned and roadmap forward

**DOI:** 10.1038/s41386-023-01667-4

**Published:** 2023-08-07

**Authors:** Mark A. Frye, Charles B. Nemeroff

**Affiliations:** 1 Department of Psychiatry & Psychology, Mayo Clinic, Mayo Clinic Alix School of Medicine, Rochester, MN USA; 2 Department of Psychiatry & Behavioral Sciences, University of Texas at Austin Dell Medical School, Austin, TX USA

**Keywords:** Neuroscience, Psychology

## Abstract

Pharmacogenomic technology is a developing field with enthusiastic interest and broad application potential. Three large, controlled studies have been published exploring the benefit of pharmacogenomically guided antidepressant treatment selection. Though all three studies did not show significant benefit of using this technology, these studies laid the foundation for further research that should address the limitations of this previous research and currently available commercial platforms. Future research needs to include large scale pharmacogenomic trials with GWAS analytics across diverse groups with attention to cost-effectiveness models, particularly for cases of treatment resistance and polypharmacy. The application of results from these large scale pharmacogenomic trials must also include exploring optimal EHR user interface design.

## Introduction

Pharmacogenomic decision support tools are an evolving technology that help clinicians make informed decisions, based on clinically actionable genetic variants in pharmacokinetic (PK) and or pharmacodynamic (PD) genes that potentially impact safety, tolerability, and response to medications. The potential value add to the index episode of care is treatment selection based on the patient’s biology, not solely relying on FDA indication or off label clinical experience. Most commercially available platforms span multiple PK and PD genes and multiple classes of psychotropic drugs (antidepressant, antipsychotics, stimulants, mood stabilizers). However, antidepressant selection for treatment of major depressive disorder has been the area of most focused research in psychiatry.

## Major studies in the field

The National Network of Depression Center’s GUIDED Trial (Genomics Used to Improve Depression Decisions) enrolled 1167 outpatients in an 8-week, randomized, patient- and rater-blind controlled study of clinician *GUIDED* antidepressant treatment selection vs treatment as usual (TAU) [1]. There was no difference in the primary outcome measure of mean change in Hamilton Depression Rating Scale (27.2% vs 24.4%, *p* = 0.107) for those participants who completed the study through week 8. Although the utility of secondary outcome measures in the context of a failed primary outcomes measure is of questionable validity [2], secondary outcome measures of response (26% vs 20%, *p* = 0.013) and remission (15% vs 10%, *p* = 0.007) favored GUIDED.

The PRIME CARE Randomized Trial enrolled 1944 patients receiving care at VA medical centers in a 24-week randomized open-label trial of pharmacogenomic guided treatment selection (*n* = 966) vs TAU (*n* = 978) [3]. Educational materials were provided for test interpretation and consideration for prescribing low risk medications. The co-primary outcome measures were the proportion of prescriptions with a predicted drug-gene interaction written and remission of depression (PHQ-9 ≤ 5). There was a significant difference with the pharmacogenomic-guided group as they were more likely to receive a medication with a lower potential drug-gene interaction (no drug-gene vs moderate/substantial interaction). Remission rates over 24 weeks were significantly higher for pharmacogenomic care vs TAU, but not significantly higher at week 24. Perlis et al. (2020) conducted a randomized clinical trial of 304 patients with major depression and found no benefit of pharmacogenomic testing [4]. Finally, the first randomized pharmacogenomic testing of depression treatment in adolescents (*n* = 176) found no significant difference in symptom improvement or side effect burden in the pharmacogenomic vs TAU groups [5]. Thus, a comprehensive review concluded that at present time, there is little evidence to support routine use of currently available pharmacogenomic tests in predicting antidepressant response [6].

## Limitation of current commercial platforms and study design

These large, controlled studies of pharmacogenomic testing for antidepressant treatment have not shown significant benefit of this decision support technology. It is important to emphasize these investigations were not designed as placebo-controlled investigational studies such as would be anticipated for a submission, for example, to the FDA for a new drug application. Future trial design should review the merits of a “placebo like” arm, but also randomization allocation, not only on treatment resistance and comorbidity, but metabolic capacity, recognizing both P450 metabolizer genotype and additional drugs that further induce or inhibit cytochrome metabolism can impact study results [7].

While the primary outcome measure of GUIDED Trial was not met, important exploratory analyses have made a positive impact on future trial design. For example, 912 participants (439 GUIDED, 473 TAU) identified from the original intent to treat cohort at baseline, had a clinically actionable variant or gene-drug interaction categorized as “use with caution” or “used with increased caution and with more frequent monitoring” [8]. Excluding treatment selection that did not pose a gene-drug interaction (i.e., no additional value of genotyping based on treatment selection), GUIDED participants had greater mean change in HDRS than TAU (27% vs 22% *p* = 0.029) as well as response (27% vs 19%, *p* = 0.008) and remission 18% vs 11%, *p* = 0.003). A second exploratory analysis evaluated if the Hamilton-D6, which focused on core symptoms of depression, would be more sensitive to change when exploring a potential biologic variable related to outcome [9]. The HAM-D6 as a continuous measure demonstrated significantly greater percentage decrease at week 8 in the GUIDED group (4.4%, *p* = 0.023); this difference was not present when using the full HDRS (3.2%, *p* = 0.069).

One could argue, given the substantial number of pharmacokinetic (PK) and pharmacodynamic genetic variants, as well as varied treatment selection (GUIDED included 21 antidepressants and 7 antipsychotics, PRIME CARE included 1406 participants who received an antidepressant), these studies were likely underpowered to identify a significant treatment arm difference. One approach to reduce this heterogeneity would be to consider a narrower group of variants or treatment(s), studying biotransformation (PK) and dynamic mechanism of drug action (PD) together. For example, a secondary analysis completed from the STAR*D study focused on depressed participants who prospectively did not achieve remission from the SSRI citalopram and were subsequently enrolled in an SNRI trial of venlafaxine [10]. Remission with venlafaxine, a 2D6 metabolized antidepressant, in prior prospective confirmation of non-remission with citalopram, a 2C19 metabolized antidepressant, was associated with a significant interaction between P450 2D6 ultra-rapid metabolizer phenotype and a greater amount of serotonin *SLC6A4 L/L* and noradrenergic *SLC6A2* transporter protein. Single drug studies targeting PK-PD interaction will have greater statistical power and clearly can provide potential clinical value in practice.

From a pharmacokinetic perspective, the bar or level of evidence for safety parameters, such as FDA black box warnings for 2D6 poor metabolizer phenotype and QT prolongation or reduced metabolic conversion of prodrug tamoxifen to chemotherapeutically active endoxifen, is significantly lower than a genetic variant that is a biomarker of treatment response. It is of note that pharmacokinetic variation has contributed to the newly FDA drug for depression treatment, dextromethorphan bupropion combination, both from the standpoint of mechanism of action, with bupropion inhibiting 2D6 increasing dextromethorphan (a NMDA antagonist) levels, as well as clinical guidelines with reduced dosing of the drug with known poor metabolizers at 2D6 (https://www.axsome.com/auvelity-prescribing-information.pdf). From a pharmacodynamic perspective, the black box warning of HLA-B*1502 variation and carbamazepine-associated Stevens-Johnsons syndrome has impacted clinical practice; further work is encouraged to explore potential PD variants related to drug safety. For example, a recent meta-analysis of 7 studies investigating antidepressant-associated treatment emergent mania (TEM) in bipolar disorder identified a significant association between the short or s allele of the serotonin transporter *SLC6A4* and TEM (OR:1.43; 95% CI:1.0–2.06; *p* = 0.05). An important commentary from the author underscored focusing on TEM biomarkers beyond serotonin transporter, highlighting no prior work with norepinephrine or dopamine transporters, and more broadly, but not part of commercial platforms today, genomewide association studies [11]. Indeed, there are several polymorphisms including the CRH binding protein [12] and the ABCB1 gene [13].

## Conclusion

Pharmacogenomic testing is a technology that will continue to advance and inherently has potential to transform clinical practice. The roadmap to greater precision in current and future novel therapeutics should be with large scale pharmacogenomic trails employing GWAS analytics and cost-effectiveness models, in particular in cases of treatment resistance and polypharmacy. To address generalizability, future studies need to prioritize diverse groups to look at possible genetic variation by ethnic groups and optimal EHR user interface design. How genomic data is added to large scale databases focused on optimal EHR interface or conventional decision support tools is yet to be determined.
